# Pneumatization Patterns of the Sphenoid Sinus in Adult Nigerians and Their Clinical Implications

**DOI:** 10.4314/ejhs.v31i6.26

**Published:** 2021-11

**Authors:** Beryl S Ominde, Joyce Ikubor, Patrick S Igbigbi

**Affiliations:** 1 Department of Human Anatomy and Cell Biology, Delta State University, Abraka, Nigeria; 2 Department of Radiology, Delta State University Teaching Hospital, Oghara, Nigeria

**Keywords:** Anterior clinoid, pneumatization, pterygoid plate, sella turcica, skull base, sphenoid sinus

## Abstract

**Background:**

The variant pneumatization patterns of the sphenoid sinus have significant surgical implications due to their associated inconsistent neurovascular relations. This study aimed at evaluating the pneumatization patterns of the sphenoid sinus in adult Nigerians.

**Methods:**

This was a retrospective study conducted at the Radiology Department of a Tertiary Hospital in Nigeria after obtaining institutional ethical approval. Brain Computed Tomography images of 336 patients (137 females, 199 males) aged ≥20 years were studied for the variant pneumatization patterns of the sphenoid sinus. Statistical Package for Social Sciences version 23 was used for data analysis. Chi-square test was used to assess for the association of the variants with gender and side. Pvalue was considered significant at <0.05.

**Results:**

The predominant pneumatization pattern in relation to the seller turcica was the sellar type (181;53.9%) followed by the presellar type (65;19.3%), post-sellar (62;18.5%), and lastly the conchal type (28;8.3%). The most prevalent clival recess was the subdorsal type (25;7.4%) followed by the dorsal (18;5.4%), combined (7;2.1%), and lastly occipital (3;0.9%). The frequency of pneumatized anterior clinoid process, greater wing of sphenoid and pterygoid process was 76;22.6%, 60;17.9% and 141;42% respectively and these showed significant side difference (P=0.001 each). None of the pneumatization patterns showed a significant gender difference. Sphenoid sinus agenesis was not observed.

**Conclusion:**

The pneumatization patterns in our study varied from the findings in previous Nigerian studies and other populations. There is therefore the need for preoperative evaluation before endoscopic transsphenoidal surgical procedures.

## Introduction

The compound unpaired bone of the anterior and middle skull base is called the sphenoid bone. It has various parts namely; a body, two pairs of butterfly-shaped wings (lesser and greater) projecting laterally from the body, and two pterygoid processes projecting inferolaterally (1). The body is aerated forming the sphenoid sinus of varying sizes and shapes among individuals. This sinus is the most inaccessible and most variant paranasal sinus whose pathologies are difficult to diagnose due to its deep location (2,3). The sinus exists at birth as a small mucosal sac of red marrow that evaginated posteriorly from the nasal capsule at the sphenoethmoidal recess during the 3^rd^ to 4^th^ month of intrauterine life (4). Pneumatization begins at 3years of age with gradual progression posteriorly and reaches the sellar floor by 7 years (3). Thereafter, aeration of the sinus proceeds rapidly and reaches its maximum size in adulthood (5). With advancing age, the sphenoid sinus enlarges due to thinning and resorption of its bony walls (1). Its pneumatization pattern varies in different races, ethnic groups, and geographical locations (6).

The sphenoid sinus is bounded by the hypophyseal fossa superiorly, the nasopharynx inferiorly, the cavernous sinus laterally, the ethmoidal sinus anteriorly, and the brainstem posteriorly (1,7). It is abutted by vital nerves and vessels such as the internal carotid artery and optic nerve whose positions in relation to the sphenoid sinus vary due to high interindividual variability in its pneumatization (8,9). The aeration ranges from minimal to extensive with potential involvement of the nearby structures that produce variant symptoms associated with sinus pathology. Additionally, the engrossed neurovascular structures pose a challenge during functional endoscopic sinus surgery (FESS) and skull base neurosurgery due to the high risk of iatrogenic injury (1). Computed Tomography (CT) is largely accepted as the imaging modality of choice in the evaluation of the morphological and volumetric characteristics of the sphenoid sinuses (9). This study therefore aimed at evaluating the pneumatization patterns of the sphenoid sinus in adult Nigerians. The imaging findings will guide surgeons in the planning of safe surgical techniques to curtail iatrogenic complications (10).

## Materials and Methods

We conducted this retrospective cross-sectional study at the Radiology Department of Delta State University Teaching Hospital, Oghara, Nigeria. This study was approved by the Hospital's Health Research and Ethics committee (Approval number; EREC/PAN/2020/030/0371). The data collection was in accordance with the ethical standards laid down by the hospital. Using purposive sampling technique, brain Computed Tomography (CT) images of adult patients taken between 1^st^ of June 2015 and 30^th^ of June 2020 were retrieved from the Picture Archiving and Communication Systems (PACS). Images of 336 patients (137 females and 199 males) aged 20 years and above who had complained of headache or with suspected stroke, brain tumors and pulmonary embolism were utilized in this study. The 20 years' age cut-off was used since the sphenoid sinus reaches its almost definitive dimensions at 20 years (11). We excluded images of patients aged below 20 years, images with the presence of craniofacial trauma, lesions of the maxillofacial area and history of surgery. Furthermore, poor-quality images with inadequate exposure, artifacts such as motion and metal artefacts, or rotation of patients were excluded.

The brain CT scans were taken using a 64 slice CT scanner (Toshiba Aquillon, Japan) at 120kV, 300mA and field of view (FOV) of 250mm. The standard operating procedure of the study center entails the acquisition of 5mm axial sections which are adequate for the evaluation of the brain, sinonasal and skull base structures, however, thinner sections of 3 mm were used to identify the minute details. Using the acquired axial CT images, multiplanar coronal and sagittal images were reformatted using 1.5mm slice thickness and 1.5mm table increment as the reconstruction parameters. A single consultant radiologist with nine years' experience evaluated the sphenoid sinus on bone window. The pneumatization patterns of the sphenoid sinus were described based on the criteria explicated by Hiremath et al (4). Using the sagittal sections, the pneumatization was classified as conchal, presellar, sellar and post-sellar and this was in relation to the anterior and posterior walls of the hypophyseal fossa. Extension of pneumatization into the clivus was categorized as subdorsal, dorsal, occipital and combined. The lateral extension into the pterygoid process as well as the greater and lesser wings of the sphenoid bone were also evaluated. We used the Statistical Package for Social Sciences (SPSS) version 23 (IBM Corporation, Armonk, New York, USA) to analyze data and summarized them in frequencies. The association of these variants with gender and side of occurrence was evaluated using the Chi-square test and considered statistically significant at P<0.05.

## Results

The Brain CT images of 199 males (59.2%) and 137 females (40.8%) were analyzed in this study. These subjects had an average age of 53.29±18.18 years. The predominant pneumatization pattern in relation to the sellar turcica was the sellar type (181,53.9%) followed by the presellar type (65,19.3%), post-sellar (62,18.5%), and lastly the conchal type (28,8.3%) (Figure 1). These patterns did not show any significant association with gender (P 0.300,0.670,0.350,0.131). The gender distribution of these patterns is shown in Table 1. Table 2 shows the comparison of their prevalence in literature. The aeration of the clivus was seen in 53 (15.8%) patients. The most common clival recess was the subdorsal type (25,7.4%) followed by the dorsal (18,5.4%), combined (7,2.1%), and lastly occipital (3,0.9%). The gender differences in the frequencies of the clival pneumatization types were not statistically significant (P 0.350,0.510, 0.790, 0.370) (Table 1).

**Table 1:** Pneumatization patterns of the sphenoid sinus

Variant pneumatization	Total		Female		Male		P value

	N	%	N	%	N	%	
**In relation to sella Turcica**							
Conchal							
Pre-sellar	28	8.3	14	10.2	14	7	0.300
Sellar	65	19.3	25	18.2	40	20.1	0.670
Post-sellar	181	53.9	78	56.9	103	51.8	0.350
	62	18.5	20	14.6	42	21.1	0.131
**Pneumatization of the clivus**							
Subdorsal	25	7.4	8	5.8	17	8.5	0.350
Dorsal	18	5.4	6	4.4	12	6.0	0.510
Occipital	3	0.9	1	0.7	2	1.0	0.790
Combined	7	2.1	4	2.9	3	1.5	0.370

**Table 2:** Population comparison of the sphenoid sinus pneumatization in relation to the sella turcica

Authors	Country	Imaging	N	Type of pneumatization %			

				Conchal	Presellar	Sellar	Post sellar
Degaga et al. (3)	Ethiopia	CT	200	2	25.5	50	22.5
Hiremath et al. (4)	India	CT	500	0	1.2	98.8	-
Yekpe et al. (6)	Benin	CT	225	0.4	24.9	74.7	-
Famurewa et al. (12)	Nigeria	CT	320	1.9	1.2	56.6	40.2
Abdalla (13)	Iraq	CT	250	0	11.2	14	74.8
Bilgir and Bayrakdar (14)	Turkey	CBCT	128	2.3	3.9	35.9	57.8
Bala and Shahdad (15)	India	CT	200	1	9	90	-
Lakshman et al. (16)	India	CT	52	0	11.5	88.5	-
Dafalla et al. (17)	Sudan	CT	506	2	21	54.7	22.3
Current study	Nigeria	CT	336	8.3	19.3	53.9	18.5

CT- Computed Tomography, CBCT- Cone Beam Computed Tomography

**Figure 2:**
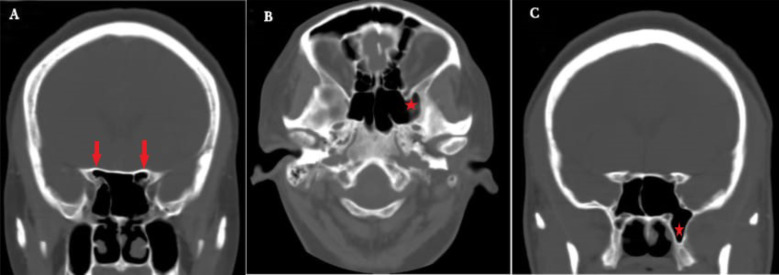
CT images showing extension of pneumatization of the sphenoid sinus. A. Bilateral anterior clinoid process pneumatization B. Left greater wing of sphenoid pneumatization C. Left pterygoid process pneumatization.

The prevalence of pneumatization of the anterior clinoid process was 76,22.6%, with a higher unilateral (45,13.4%) than bilateral occurrence (31,9.2%). Involvement of the unilateral left and right anterior clinoid process was seen in 20,6% and 25,7.4% patients respectively. The prevalence of pneumatization of the greater wing of sphenoid was 60,17.9% with more unilateral (42, 12.5%) than bilateral existence (18, 5.4%). Unilateral aeration of the greater wing was more frequent on the left (24,7.1%) than on the right (18,5.4%). The frequency of pneumatized pterygoid process was 141 (42%), with unilateral occurrence in 63 (18.8%) patients, and more common on the left (44,13.1%) than on the right (19,5.7%). The occurrence of bilateral pterygoid process pneumatization was 78 (23.2%) (Fig.2). The prevalence of the anterior clinoid process, greater wing and pterygoid process pneumatization showed significant side differences (p=0.001 each). However, their occurrence was not associated with gender (Table 3). We did not observe sphenoid sinus agenesis in the images assessed (0,0%).

**Table 3:** Extension of pneumatization of the sphenoid sinus

Site		Total		Female		Male		P value

		N	%	N	%	N	%	
Anterior clinoid process	Right	56	16.7	23	16.8	33	16.6	0.960
	Left	51	15.2	19	13.9	32	16.1	0.580
Greater wing of sphenoid	Right	36	10.7	15	10.9	21	10.6	0.910
	Left	42	12.5	16	11.7	26	13.1	0.710
Pterygoid process	Right	97	28.9	47	34.3	50	25.1	0.068
	Left	122	36.3	57	41.6	65	32.7	0.094

## Discussion

The predominant pneumatization pattern was the sellar type (53.9%) whose frequency was comparable to 56.6% documented in Osun state, Nigeria (12). Our findings were higher than those reported in Turkey and Iraq and lower than the prevalence in India (4,13,14). The frequency of the presellar type, 19.3% was higher than 1.2% reported in a previous Nigerian study by Famurewa et al. (12) According to literature reports, the frequency of the presella type was higher in Ethiopians (25.5%) and lower in Turkish (3.9%), and Indians (9%) (3,14,15). The conchal type had a low prevalence (8.3%) although this was higher than some previously documented reports (3,12,15). It was absent in the populations studied by Hiremath et al., Lakshman et al. and Abdalla (4,13,16) (Table 2). The prevalence of the post-sellar type was lower than the findings documented by previous African studies (3,12,17). Studies from Iraq and Turkey also reported higher prevalence of the post-sellar pneumatization of the sphenoid sinus (13,14).

The variability in the extent of pneumatization in different literature reports is credited to the discrepancies in imaging techniques with varying thickness of CT slices, differences in the definition of each type as well as ethnicity and race (6). We also suggest the contribution from geographical, genetic, and environmental factors. The knowledge of the variant pneumatization of the sphenoid sinus in relation to the sellar turcica is important to surgeons. The sellar type facilitates expanded endoscopic approach (EEA) with easy identification of anatomical landmarks hence low risk of injury to neurovascular structures. It also requires less bone removal for better surgical exposure. The anterior wall of the presellar type can be opened to allow exploration of the base of sellar turcica. The conchal type poses a great challenge during endoscopic management of sphenoid sinus, pituitary, and anterior skull base pathology since they require drilling and removal of the thick cancellous bone (15).

Clival pneumatization had a low prevalence of 15.8%. The subdorsal type was the most prevalent (7.4%) followed by the dorsal, combined, and lastly occipital (5.4%, 2.1%, 0.9%). The frequency of the pneumatized dorsum sella in Nigerians of Osun state was 1.6% slightly lower than 2.1% in our study (12). The occurrence of the subdorsal, dorsal, occipital, and combined clival pneumatization patterns were higher in the Indians (84.8%, 5.2%, 5%, 5%) studied by Hiremath et al. (4) The variations can be attributed to genetic, racial, and ethnic differences. The awareness of clival pneumatization is imperative since the thin clivus is suitable for the transnasal approach into the posterior cranial fossa. The occipital type often establishes contact with the meninges, basilar plexus, basilar artery, and pons. Sphenoid sinusitis in this region can lead to cerebrospinal fluid (CSF) fistula, basilar artery vasculitis, and ischemic infarction of the pons (8).

The prevalence of the pneumatized anterior clinoid process (22.6%) was higher than the reports in previous Nigerian studies by Fasunla et al., Onwuchekwa and Alazigha, Famurewa et al., and other studies outside Nigeria (5,6,7,12,18) (Table 4). However, Joghataei et al. and Gungor and Okur documented higher frequencies in Iran and Turkey respectively (2,19) (Table 4). The population differences could be ascribed to genetics, ethnicity, race, and environmental factors. The differences in the resolution of CT scanners used in various studies may also contribute to the variation (12). Pneumatized anterior clinoid process may be associated with the internal carotid artery (ICA) and optic nerve bulge or dehiscence which may be inadvertently injured during FESS (8). Its preoperative identification is important in patients with sellar and suprasellar masses, cavernous sinus lesions, and periclinoid aneurysms that may require anterior clinoidectomy to avoid postoperative pneumocephalus and rhinorrhea (4).

**Table 4:** Extension of sphenoid sinus pneumatization in different populations

Authors	Country	N	Pneumatization %		

			PP	ACP	GWS
Joghataei et al. (2)	Iran	129	24.8	43.4	41.1
Degaga et al. (3)	Ethiopia	200	15	18	16.5
Fasunla et al. (5)	Nigeria	110		14.5	
Yekpe et al. (6)	Benin	225	7.3	7.1	3.3
Singh et al. (7)	India	84	32.2	17.85	22.6
Famurewa et al. (12)	Nigeria	320	45.1	11.6	35
Onwuchekwa and Alazigha (18)	Nigeria	110		6.36	
Gungor and okur (19)	Turkey	132	44.1	28.8	21.9
Dasar and Gokce (21)	Turkey	400	51.7	25.5	18.2
Current study	Nigeria	336	42	22.6	17.9

PP- pterygoid process, ACP- anterior clinoid process, GWS-greater wing of sphenoid

The occurrence of an aerated greater wing of sphenoid (17.9%) was lower than the findings from a previous Nigerian study (Osun state) by Famurewa et al. as well as reports from Sudan and Iran (2,12,20). However, the frequency was higher in the West Africans of Benin (6) (Table 4). The prevalence of pterygoid process aeration was 42%, almost similar to 40.3%, 44.1%, and 45.1% reported by Kajaok et al., Gungor and Okur, and Famurewa et al. respectively (12,19,20). A higher frequency was reported by Dasar and Gokce while Yekpe et al. documented a lower prevalence (6,21). The discrepancy in the prevalence of aerated greater wing and pterygoid process in literature may perhaps be due to genetic, ethnic, and racial differences. Pneumatization of the greater wing of sphenoid and pterygoid process is important to surgeons since it renders the maxillary nerve, vidian nerve, and ICA susceptible to iatrogenic injury and neuralgia in sphenoid sinusitis (4). The endoscopic endonasal transpterygoid approach to the central skull base uses a pneumatized pterygoid process to access the middle and posterior skull base for biopsy of lesions or repair of CSF leaks (7,19).

None of the CT images evaluated in this study had sphenoid sinus aplasia. This was consistent with the reports by Papadopoulou et al. who documented that this variant is rare (8). Dasar and Gokce documented a low prevalence of 2.5% in Turkey (21). The knowledge of the existence of sphenoid sinus aplasia is important to avoid its misdiagnosis as sinusitis or neoplasm. A non-pneumatized sphenoid sinus may be associated with craniofacial anomalies or skeletal diseases. Furthermore, preoperative identification of sphenoid sinus agenesis is important before FESS (15).

In conclusion, the pneumatization patterns in our study varied from the findings in previous Nigerian studies and other populations. There is therefore the need for preoperative evaluation before endoscopic transsphenoidal surgical procedures.

The findings of this study may not be generalized since we used the purposive sampling technique and analyzed CT images from a single center. Additionally, the analysis was performed on brain CT images and not maxillofacial CT images whose axial cuts are thinner and with better resolution.

**Future research**

We recommend a study using a larger sample size of the CT images from multiple centers in Nigeria to confirm our findings.

## References

[1] Kusch AM, Garcia VR (2019). Giant pneumatization of sphenoid sinus: Report of four cases and review of literature. Rev Med Hered.

[2] Joghataei MT, Hosseini A, Ansari MJ, Golchini E, Namjoo Z, Mortezaee K (2019). Variations in the anatomy of sphenoid sinus: A Computed Tomography investigation. J Pharm Res Int.

[3] Degaga TK, Zenebe AM, Wirtu AT, Woldehawariat TD, Dellie ST, Gemechu JM (2020). Anatomographic Variants of Sphenoid Sinus in Ethiopian Population. Diagnostics.

[4] Hiremath SB, Gautam AA, Sheeja K, Benjamin G (2018). Assessment of variations in sphenoid sinus pneumatization in Indian population: A multidetector computed tomography study. Indian J Radiol Imaging.

[5] Fasunla AJ, Ameye SA, Adebola OS, Ogbole G, Adeleye AO, Adekanmi AJ (2012). Anatomical variations of the sphenoid sinus and nearby neurovascular structures seen on computed tomography of black Africans. East Cent Afr J Surg.

[6] Yekpe P, Akanni D, de Souza CO, Adjadohoun S, Kiki M, de Tove KMS (2018). anatomic variants of sphenoid sinuses and adjacent structures: a study of 225 skull CT Scans at CNHU-HKM in Benin, West Africa. Open J Radiol.

[7] Singh BP, Metgudmath RB, Singh D, Saxena U (2019). Anatomical variations of sphenoid sinus among patients undergoing computed tomography of paranasal sinus. Int J Otorhinolaryngol Head Neck Surg.

[8] Papadopoulou A, Chrysikos D, Samolis A, Tsakotos G, Troupis T (2021). Anatomical Variations of the Nasal Cavities and Paranasal Sinuses: A Systematic Review. Cureus.

[9] Gibelli D, Cellina M, Gibelli S, Oliva AG, Codari M, Termine G, Sforza C (2018). Volumetric assessment of sphenoid sinuses through segmentation on CT scan. Surg Radiol Anat.

[10] Cellina M, Gibelli D, Floridi C, Toluian T, Valenti Pittino C, Martinenghi C, Oliva G (2020). Sphenoid sinuses: pneumatisation and anatomical variants-what the radiologist needs to know and report to avoid intraoperative complications. Surg Radiol Anat.

[11] Baldea V, Sandu OE (2012). CT study of the sphenoid sinus pneumatization types. Romanian J Rhin.

[12] Famurewa OC, Ibitoye BO, Ameye SA, Asaleye CM, Ayoola OO, Onigbinde OS (2018). Sphenoid sinus pneumatization, septation and the internal carotid artery: A computed tomography study. Niger Med J.

[13] Abdalla MA (2020). Pneumatization patterns of Human sphenoid sinus associated with internal carotid artery and optic nerve by CT scan. Ro J Neurol.

[14] Bilgir E, Bayrakdar I (2021). A new classification proposal for sphenoid sinus pneumatization: a retrospective radio-anatomic study. Oral radiol.

[15] Bala SS, Shahdad S (2019). Gender distribution and clinical significance of pneumatization in sphenoid sinus. Int J Contemp Med Res.

[16] Lakshman N, Viveka S, Thondupadath AF (2020). Anatomical relationship of pterygoid process pneumatization and vidian canal. Braz J Otorhinolaryngol.

[17] Dafalla SE, Seyed MA, Elfadil NA, Elmustafa OM, Hussain Z (2017). A Computed Tomography-aided clinical report on anatomical variations of the paranasal sinuses. Int J Medical Res Health Scie.

[18] Onwuchekwa RC, Alazi N (2017). Computed tomography anatomy of the paranasal sinuses and anatomical variants of clinical relevant in Nigerian adults. Egypt J Ear Nose Throat Allied Sci.

[19] Gungor G, Okur N (2019). Evaluation of paranasal sinus variations with computed tomography. Haydarpasa Numune Med J.

[20] Kajoak SA, Ayad CE, Najmeldeen M, Abdalla EA (2014). Computerized Tomography morphometric analysis of the sphenoid sinus and related structures in Sudanese population. Global Advanced Research J of Med and Medical Scie.

[21] Dasar U, Gokce E (2016). Evaluation of variations in sinonasal region with computed tomography. World J Radiol.

